# Statistics of Medical Schools

**Published:** 1846-10

**Authors:** 


ARTICLE VIII.

### STATISTICS OF MEDICAL SCHOOLS.

We have received the Catalogues and Announcements of a number of Medical Institutions, from which it would appear that the number in attendance during the last session, and of those who have entered the ranks of the profession, will compare well with those of previous years. A general effort seems to have been made to increase the means of instruction for the coming session, each Institution vieing with the others in presenting to students facilities for study as the best means of inducing attendance. We give below a list of the Institutions and the numbers in attendance upon the lectures of 1845-46, and the graduates of '46, as far as we have been able to learn.

| | Students. | Graduates. |
|---|---|---|
| Medical College of Georgia, | 112 | 33 |
| University of Pennsylvania, | 462 | 168 |
| Jefferson Medical College, | 469 | 170 |
| Transylvania University, | 171 | 64 |
| Albany Medical College, | 115 | 42 |
| Louisville Medical Institute, | 245 | 78 |
| Willoughby Medical College, | 164 | 30 |
| Medical College of Louisiana, | 103 | 19 |
| Geneva Medical College, | 178 | |
| Medical School of Maine, | 73 | 19 |
| Harvard University, | 159 | 31 |
| Yale College, (Medical Institution,) | 53 | 20 |
| University of the City of New York, | 407 | 131 |
| College of Physicians and Surgeons, (N.Y.) | 200 | 38 |
| Western Reserve College, (Cleaveland,) | 160 | 50 |
| Berkshire Medical Institution, | 142 | 35 |
| University of the State of Missouri, | 92 | 29 |
| St. Louis University, | | 11 |

| | | |
|---|---|---|
| Medical College of Ohio, | 195 | 46 |
| Castleton Medical College, | 140 | 36 |
| Rush Medical College, | 50 | 10 |
| Maryland University, | 147 | 40 |
| Indiana Medical College, | 81 | 18 |
| Medical College of South Carolina, | 214 | 74 |
| Pennsylvania Medical College, | 94 | 38 |
| Vermont Medical College, | | 24 |

